# Willingness to take long-acting injectable pre-exposure prophylaxis among men who have sex with men who participated in the CROPrEP study: a cross-sectional online study

**DOI:** 10.1186/s12889-023-17325-9

**Published:** 2023-12-13

**Authors:** Yingjie Liu, Zhenxing Chu, Hongyi Wang, XiaoJie Huang, YaoKai Chen, Hui Wang, Dehua Zou, YongJun Jiang, WenQing Geng, Qinghai Hu, Baosen Zhou, Hong Shang

**Affiliations:** 1 NHC Key Laboratory of AIDS Immunology, National Clinical Research Center for Laboratory Medicine, China Medical University, The First Hospital of China Medical University, No. 155 Nanjing N St, Heping District, Shenyang, Liaoning Province 110001 China; 2 Key Laboratory of AIDS Immunology of Liaoning Province, The First Hospital of China Medical University, Shenyang, Liaoning, China; 3 Key Laboratory of AIDS Immunology, Chinese Academy of Medical Sciences, Shenyang, Liaoning, China; 4 Infectious Disease Department, Beijing Youan Hospital, Capital Medical University, Beijing, China; 5 Chongqing Public Health Medical Center, Chongqing, China; 6 Department of Infectious Diseases, Shenzhen Third People’s Hospital, Shenzhen, China; 7 Department of Clinical Epidemiology, The First Hospital of China Medical University, No. 155 Nanjing N St, Heping District, Shenyang, Liaoning Province 110001 China

**Keywords:** Men who have sex with men, HIV, Long-acting injectable, Pre-exposure prophylaxis, Willingness, Factor

## Abstract

**Introduction:**

Evidence on the willingness of men who have sex with men (MSM) with oral pre-exposure prophylaxis (PrEP) experience, especially those with suboptimal adherence, to take long-acting injectable PrEP (LAI-PrEP) is critical to guide future LAI-PrEP implementation.

**Objective:**

The objective was to assess the willingness of MSM with oral PrEP experience to take LAI-PrEP.

**Methods:**

MSM who participated in the China Real-world Study of Oral PrEP (CROPrEP) were enrolled in this study. Information on the willingness of MSM to take LAI-PrEP and potential correlates was collected using a structured online questionnaire. The main outcomes were the willingness of MSM to take LAI-PrEP and its association with HIV-related behaviours, sexually transmitted infections, and oral PrEP history. Logistic regression was used to identify correlates of the willingness of MSM to take LAI-PrEP.

**Results:**

A total of 612 former CROPrEP participants (FCPs) were included in this study. There were 315 (51.5%) daily oral PrEP (D-PrEP) users and 297 (48.5%) event-driven oral PrEP (ED-PrEP) users at the last follow-up. Most FCPs (77.8%) were willing to take free LAI-PrEP. FCPs with no less than two sexual male partners (aOR = 1.54, [95% CI: 1.04, 2.29], *P* = 0.031), those with male partners with unknown HIV statuses (aOR = 2.04, [95% CI: 1.31, 3.18], *P* = 0.002), those with recreational drug use (aOR = 1.58, [95% CI: 1.05, 2.40], *P* = 0.030), and those with HSV-2 positivity (aOR = 2.15, [95% CI: 1.30, 3.57], *P* = 0.003) were more willing to take LAI-PrEP, while ED-PrEP users (aOR = 0.66, [95% CI: 0.45, 0.98], *P* = 0.037) and FCPs with suboptimal oral PrEP adherence (aOR = 0.58, [95% CI: 0.36, 0.94], *P* = 0.026) were less willing to take LAI-PrEP.

**Conclusion:**

LAI-PrEP has good prospects for expanding PrEP coverage. However, FCPs with suboptimal oral PrEP adherence are less likely to take LAI-PrEP. Further intervention and implementation efforts are needed to improve the willingness of MSM to use LAI-PrEP, and sexual health should be considered during the discussion about PrEP initiation.

**Supplementary Information:**

The online version contains supplementary material available at 10.1186/s12889-023-17325-9.

## Introduction

Pre-exposure prophylaxis (PrEP) is recommended as a novel biomedical human immunodeficiency virus (HIV) prevention tool for key populations [1]. The burden of HIV is disproportionately high among men who have sex with men (MSM) [2]. Multiple randomized controlled trials and real-world studies have shown that daily PrEP (D-PrEP) and event-driven PrEP (ED-PrEP) can effectively reduce the risk of HIV transmission among high-risk MSM [3]. According to HIV surveillance, as one of the first areas to implement oral PrEP, San Francisco has seen a downward trend in HIV incidence since the implementation of PrEP [4]. As of March 2022, oral PrEP has been approved in 87 countries and territories worldwide to prevent HIV infection in key populations [5]. In China, the implementation and promotion of PrEP have reached a pivotal stage. The first real-world PrEP demonstration project (China Real-world Study of Oral PrEP [CROPrEP]) has been completed. The study findings indicated that both D-PrEP and ED-PrEP regimens are associated with a reduced incidence of HIV among high-risk MSM [6]. Furthermore, the initial version of the *HIV PrEP Experts Consensus* was published in 2020 [7]. Additionally, the 2021 edition of the Chinese guidelines for the diagnosis and treatment of HIV/AIDS provided comprehensive recommendations for PrEP, emphasizing its recommendation for key populations, including MSM, transgender individuals and sex workers [8]. The target population for PrEP can seek services at accredited health care facilities or designated infectious disease hospitals across different regions. They undergo a comprehensive evaluation conducted by a qualified health care professional and subsequently obtain PrEP at their own expense for HIV prevention purposes.

Oral PrEP efficacy is strongly associated with medication adherence. When MSM have good adherence, oral PrEP can reduce their risk of HIV infection [3]. When medication adherence decreases to 51%, the efficacy of oral PrEP decreases to 44% [9]. A recent meta-analysis showed that a one-ten percent decrease in oral PrEP adherence decreases its efficacy by 13% [10]. The key barriers to oral PrEP adherence are related to three ecological levels: the individual (e.g., low awareness of PrEP or HIV susceptibility and forgetfulness), interpersonal (e.g., nonsupport from family and peers and stigma), and structural (e.g., lack of health insurance coverage and low-quality oral PrEP services) levels [11–13]. Multilevel interventions have been proposed to promote oral PrEP adherence and persistence [11]. However, despite the effectiveness of PrEP and recent efforts in promoting PrEP adherence, oral-PrEP users have been shown to have suboptimal adherence, and over four in ten discontinue PrEP prematurely [13].

Long-acting injectable PrEP (LAI-PrEP) may hold promise in solving some disadvantages of oral PrEP in terms of access, retention, and adherence [14, 15]. A previous study revealed that intramuscularly long-acting injectable cabotegravir (CAB-LA) every 8 weeks was superior to daily oral TDF-FTC in preventing HIV infection among MSM [16]. The United States Food and Drug Administration (FDA) approved LAI-PrEP for HIV prevention in December 2021 [17]. In China, the CAB injection agent was approved for HIV treatment but has not been approved for HIV prevention [18]. Large-scale studies have shown that MSM have a high willingness to take LAI-PrEP and even prefer LAI-PrEP to oral PrEP [19–21]. LAI-PrEP is popular among MSM who have oral PrEP experience, and there is potential for current oral PrEP users to switch to LAI-PrEP [22–24]. The availability of LAI-PrEP needs to be expanded especially among oral PrEP users with poor medication adherence to yield increased population coverage of PrEP [14, 15]. The willingness and preferences for LAI-PrEP in samples of MSM with or without oral PrEP experience have been assessed. However, few studies have assessed the willingness of potential LAI-PrEP-targeted MSM with poor oral PrEP adherence. In addition, there is little evidence about the factors related to LAI-PrEP willingness among MSM with oral PrEP experience.

Thus, in this study, we investigated the willingness to take LAI-PrEP and factors influencing willingness among MSM who completed the CROPrEP demonstration project considering oral PrEP adherence to provide an update of factors influencing LAI-PrEP willingness.

## Methods

### Study design and participant collection

This study was a cross-sectional survey that enrolled participants of the CROPrEP demonstration project from four large Chinese cities (Beijing, Shenyang, Shenzhen, and Chongqing) between December 2018 and October 2019 and followed them up for 12 months. The inclusion criteria of the CROPrEP project were as follows: (1) men aged 18 to 65 years who were HIV seronegative, (2) men who had engaged in oral and/or anal intercourse with men at any point in their lives and (3) men who reported at least one sexual risk factor in the past 6 months, such as engaging in condomless receptive anal intercourse (CRAI) with men, having two or more male sexual partners, having a history of sexually transmitted infections (STIs), or using post-exposure prophylaxis [6]. At the conclusion of the CROPrEP project in October 2020, participants who attended the 12-month visit and provided consent were invited to participate in this cross-sectional survey study; participants were recruited from November 2020 to January 2021 (registration number ChiCTR2000038416). The enrolment criteria were as follows: (1) participants who completed the 12-month follow-up of the CROPrEP project and (2) those who were willing to provide informed consent to participate in this study.

Community-based organization (CBO) workers released recruitment information, including the survey aims and producers, the participants’ rights and duties, and ways to participate via preestablished WeChat groups, which were used to contact former CROPrEP participants (FCPs). For FCPs who did not immediately complete the questionnaire, CBO workers sent recruitment information three times to ensure more responses. FCPs who were willing to participate in this study could click the website link or scan the preregistered Quick Response Code to sign an electronic informed consent form (Supplement 1) and then respond to an anonymous online survey (Supplement 2). Each participant was allowed to access the online survey only once. The respondents received a 20-yuan (approximately US $3.1) honourarium after completing the questionnaire. This study was approved by the Institutional Review Board at the First Hospital of China Medical University ([2020]-327). The Checklist for the Reporting of Observational Studies in Epidemiology (STROBE) Statement (Table 1 in Supplement 3) and Reporting Results of Internet E-Surveys (CHERRIES) were used for this study (Table 2 in Supplement 3).

**Table 1:** Socio-demographic characteristics, HIV risk behaviors and among former CROPrEP participants (*n* = 612)

Characteristics	Number (all = 612)	Percent (%)
Socio-demographic characteristics		
Age groups (years)		
18–29	243	39.7
30–49	326	53.3
50 or above	43	7.0
Education level		
High school or less	108	17.6
College and greater	504	82.4
Monthly personal income, CNY (USD)		
No fixed income	69	11.3
Below 4,000 (619)	159	26.0
At least 4,000 (619)	384	62.7
Housing condition		
Unstable	10	1.6
Relatively stable	602	98.4
HIV-related behaviors in the past 3 months		
≥ 2 sex partners with anal sex	359	58.7
CRAI with male sex partners	205	33.5
HIV positive male sexual partners		
No	351	57.4
Yes	47	7.7
Not sure	214	35.0
Recreational drug use	257	42.0
History of STIs		
Syphilis Positive		
No	492	80.4
Yes	120	19.6
HSV-2 Positive		
No	446	72.9
Yes	166	27.1
CROPrEP history		
Oral PrEP regimen		
Daily	315	51.5
Event-driven	297	48.5
Switched oral PrEP regimen		
Yes	183	29.9
No	429	70.1
Suboptimal oral PrEP adherence		
No	504	82.4
Yes	108	17.6

*Note*: *CNY* Chinese Yuan, *USD* United States dollar, *HIV* human immunodeficiency virus, *CRAI* condomless receptive anal intercourse, *STI* sexual transmitted infection, *HSV-2* herpes simplex virus 2, *CROPrEP* the China Real-world Study of Oral PrEP, *PrEP* pre-exposure prophylaxis

**Table 2:** willingness and preference to LAI-PrEP and oral PrEP

Characteristics	Number (all = 612)	Percent (%)
Hear about LAI-PrEP		
Yes	219	35.8
No or not sure	393	64.2
Free LAI-PrEP willingness		
Yes	476	77.8
No or not sure	136	22.2
LAI-PrEP payment willingness		
Yes	250	40.8
No or not sure	362	59.2
Preference		
LAI-PrEP	203	33.2
Oral PrEP	90	14.7
The more effective way	121	19.8
Either or not sure	196	32.0
None	2	0.3

*Note*: *PrEP* pre-exposure prophylaxis, *LAI-PrEP* long-acting injectable PrEP

### Data collection

The information collected from the online questionnaire included sociodemographic information, HIV risk behaviours in the last three months, and PrEP-related willingness and preference for PrEP modalities (Supplement 2). The sociodemographic characteristics included age, education level, employment status, monthly income, and housing conditions. HIV risk behaviours in the past three months included the numbers of anal sex partners, HIV-positive male partners, CRAI with male partners and recreational drug use. The willingness of the participants to take LAI-PrEP (with possible answers of yes, no, and not sure) included the willingness to take free LAI-PrEP (If free LAI-PrEP was available, but you had to pay for PrEP-related routine laboratory tests, would you take LAI-PrEP to prevent HIV infection?) and pay for LAI-PrEP (If the CROPrEP group provided free counselling services, would you pay for LAI-PrEP medications and related routine laboratory tests to prevent HIV infection?). Due to the lack of awareness about LAI-PrEP among FCPs, their knowledge about LAI-PrEP was surveyed before inquiring about their willingness to take it. For FCPs who indicated that they had not heard of LAI-PrEP or were not sure, the questionnaire automatically provided a brief introduction to LAI-PrEP. Preferences regarding the two different PrEP modalities were assessed with the following possible answers: LAI-PrEP, oral PrEP, the more effective way, either, none, and not sure.

Data on the history of STIs, oral PrEP regimen and PrEP adherence were based on FCPs’ previous on-site records. An STI history included diagnoses of syphilis and herpes simplex virus 2 (HSV-2) during the CROPrEP study. At each clinical visit in the CROPrEP study, participants were asked to provide blood specimens for STI monitoring. Serological screening for syphilis was conducted using a rapid plasma reagin (RPR) test. Positive RPR results were confirmed through a Treponema pallidum particle assay (TPPA). Subjects with positive TPPA and RPR results were diagnosed with syphilis. For HSV-2 testing, if the HSV-2-IgG test was positive, further testing for HSV-2-IgM was conducted. If both tests are positive, the participant was considered to have HSV-2 [6].

The PrEP regimen referred to the method of oral PrEP. The CROPrEP project used Truvada® as oral PrEP, and participants could choose the daily (taking one pill every 24 h) or event-driven (taking oral PrEP medicine with a “2–1-1” scheme before and after performing sexual activities) PrEP regimen. Participants could switch PrEP regimens during the study period [6]. This study utilized the regimens at the time of the final follow-up visit for analysis. Furthermore, PrEP adherence referred to taking the pills as prescribed by the doctor. Data on PrEP adherence in this study were accessed by pill counting [6]. In the CROPrEP study, participants completed 5 follow-up visits at 1, 3, 6, 9, and 12 months. For each clinic visit, experienced physicians recorded both the number of medication tablets returned by the participants and the number of tablets allocated to them. The number of tablets allocated at the previous visit minus the number of medication tablets returned at the current visit represented the number of tablets consumed by participants between the two follow-up visits. For participants who chose the daily PrEP regimen, taking less than 4 tablets per week was defined as suboptimal medication adherence [25]. For participants who chose the event-driven PrEP regimen, PrEP adherence was defined as the combination of sexual behaviours and medication use. Event-driven PrEP users were recommended to take oral PrEP with a “2–1-1” scheme before and after performing sexual activities. When there was no sexual activity or risk, there was generally no need for PrEP medication. Taking less than 4 tablets per week was defined as suboptimal medication adherence for ED-PrEP users with sexual behaviours or risk [26]. This study utilized PrEP adherence for the whole follow-up period for analysis.

### Quality control

The questionnaire included two questions for quality control: a question with a wrong option (“What's the weather today? Sunny/Cloudy/Raining/Snowing/Sunday”) and a question with a predetermined answer (“You should only choose the option of “Completely agree”. Completely agree/Basically agree/Disagree”). Responses only with the correct answers to these two questions were included in the statistical analyses. Questionnaires with a response time of less than 3 min or logical errors were excluded. The response time threshold was chosen to balance data quality and the inclusion of engaged participants. Logical errors included inconsistent responses, nonsequential responses, inaccurate dates, repetitive or monotonous responses, overuse of "not sure", etc.

### Statistical analysis

Variables with > 5% missing values were excluded unless otherwise stated, and imputation was performed for variables with ≤ 5% missing values. The missing categorical variables (missing HSV-2 test result [*n* = 30] and missing syphilis test result [*n* = 1]) were used for mode imputation. Descriptive statistics were used to summarize the data characteristics. Categorical variables were described by the frequency and percentage, and continuous variables were described by the mean and standard deviation or median and interquartile range. Chi-square ($\chi 2$) tests were used to identify the differences in characteristics between the respondents and nonrespondents and the differences between respondents who had suboptimal oral PrEP adherence and those who were unwilling to take LAI-PrEP and others. Univariable logistic regression was used to calculate odds ratios (ORs) and their 95% confidence intervals (CIs) for factors associated with the willingness to take LAI-PrEP. Variables with a *P* < 0.2 in the univariate models were included in the multivariable logistic regression analysis using the “enter” method with adjustment for sociodemographic characteristics. A two-sided *P* < 0.05 was considered statistically significant. All analyses were performed using SPSS Statistics version 26.0 (IBM Corporation, USA).

## Results

**Fig. 1:**
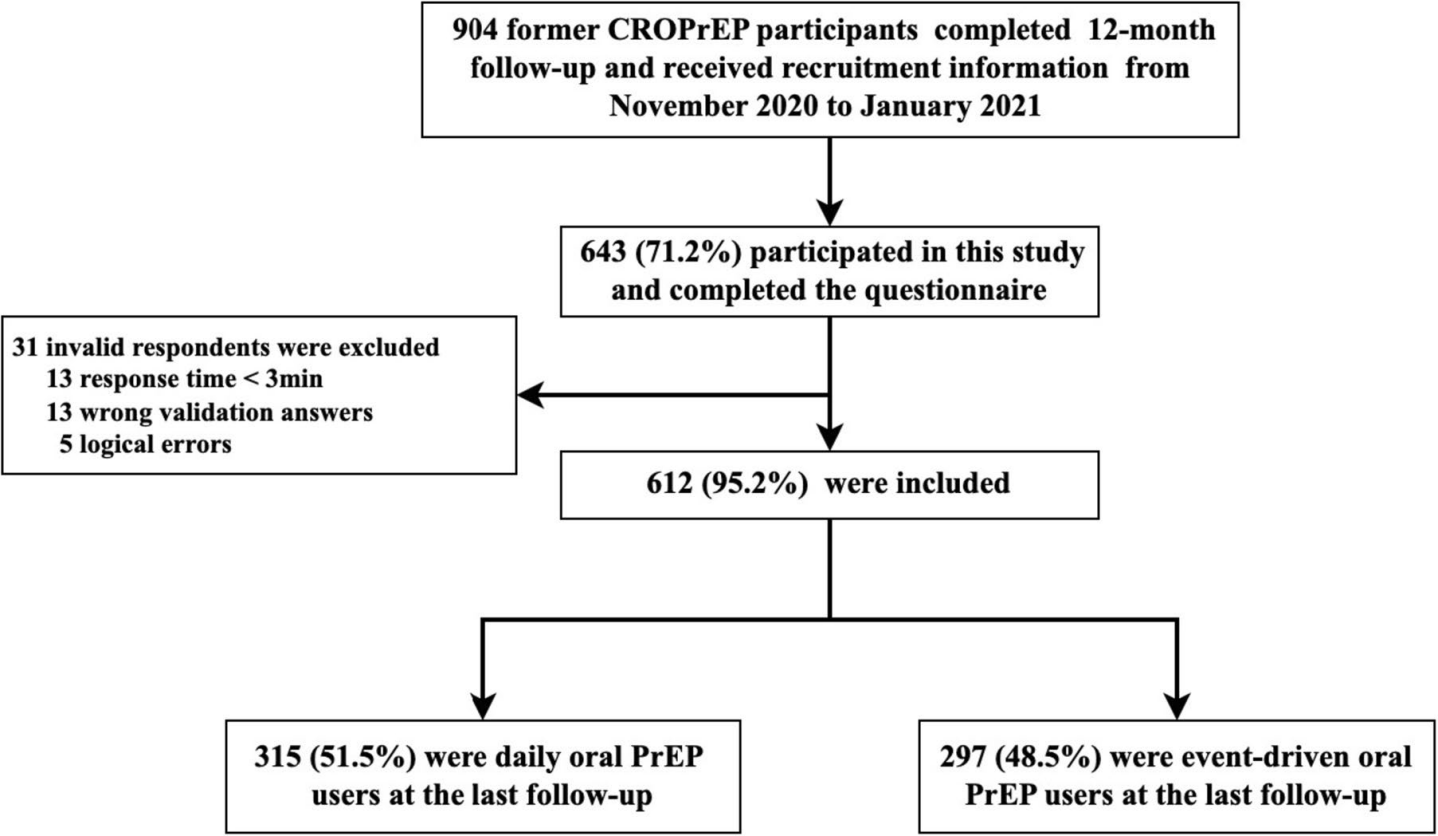
flowchart of the study procedure

A total of 904 FCPs who completed the 12-month follow-up were enrolled in this study, and 643 (71.1%) completed the final survey. In total, 31 respondents were excluded: 13 for an answering time of less than 3 min, 13 for wrong validation answers, and 5 for logical errors. Ultimately, a total of 612 (612/643, 95.2%) FCPs, including 315 (315/612, 51.5%) former D-PrEP users and 297 (297/612, 48.5%) former ED-PrEP users, were included in the final analyses (Fig. 1). Compared with MSM who completed the 12-month follow-up but were not included in this study, those included in the study had low monthly income, more D-PrEP regimens, and less switching of the oral PrEP regimen (*P* < 0.05, Table S3 in Supplement 4).

### Characteristics of participants

A total of 326 FCPs (326/612, 53.3%) were 30–49 years old, 504 (504/612, 82.4%) had a college degree or above, 384 (384/612, 62.7%) had a monthly income more than ¥4000 ($619), and 602 (602/612, 98.4%) had relatively stable housing conditions. Regarding HIV risk behaviours in the previous three months, 58.7% (359/612) had more than two male sex partners, 33.5% (205/612) had CRAI with male sex partners, 35.0% (214/612) did not know their male sexual partners’ HIV statuses and 42.0% (257/612) used recreational drugs. For the history of STIs, 19.6% (120/612) and 27.1% (166/612) of the participants had syphilis and HSV-2 positivity, respectively. In the former CROPrEP project, over half (315/612, 51.5%) of the FCPs were D-PrEP users, 29.9% (183/612) of the FCPs switched to the PrEP regimen, and 17.6% (108/612) of the FCPs had suboptimal oral-PrEP adherence during the 12-month follow-up (Table 1).

### Willingness to take PrEP and preference for PrEP

Only 35.8% (219/612) of the FCPs had heard about LAI-PrEP. After a brief introduction to LAI-PrEP, 77.8% (476/612) of all FCPs were willing to take free LAI-PrEP, and 40.8% (250/612) were willing to pay for it to prevent HIV infection. Regarding preferences for the two different PrEP modalities, nearly one-third (203/612, 33.2%) of the FCPs specifically preferred LAI-PrEP, and the remaining FCPs preferred oral PrEP (90/612,14.7%), whichever was most effective (121/612, 19.8%), either (173/612, 28.3%), had no preference (2/612, 0.3%) or were not sure (23/612, 3.8%, Table 2).

### Factors associated with the willingness to take LAI-PrEP

With adjustment for sociodemographic characteristics including age, educational background, monthly income, and housing conditions, the independent factors positively associated with LAI-PrEP willingness were as follows: having no less than two sexual male partners (vs. less than two sexual male partners, aOR = 1.54, 95% CI 1.54–2.29), not knowing male partners’ HIV statuses (vs. having HIV-negative male partners, aOR = 2.04, 95% CI 1.31–3.18), using recreational drugs (vs. no recreational drug use, aOR = 1.58, 95% CI 1.05–2.40), and HSV-2 positivity (vs. HSV-2 negativity, aOR = 2.15, 95% CI 1.30–3.57). Being a former ED-PrEP user in the CROPrEP project (vs. D-PrEP users, aOR = 0.66, 95% CI 0.45–0.98) and having suboptimal oral-PrEP adherence (vs. good oral PrEP adherence, aOR = 0.58, 95% CI 0.036–0.094) were negatively associated with the willingness to take LAI-PrEP (*P* < 0.05, Table 3).

**Table 3:** Univariable and multivariable logistic analyses of correlates associated with free LAI-PrEP use (*n* = 612)

	Univariate		Multivariate	
	OR (95%CI)	P	aOR (95%CI)	P
Socio-demographic characteristics				
Age (year)				
18–29	Ref			
30–49	1.49 (1.00, 2.22)	0.052	-	-
50 or above	0.79 (0.39, 1.61)	0.517	-	-
Education level				
High school or less	Ref			
College and greater	1.81 (1.14, 2.87)	0.012	-	-
Monthly income, CNY (USD)				
No fixed income	Ref			
Below 4,000 (619)	1.82 (0.98, 3.39)	0.058	-	-
At least 4000 (619)	2.16 (1.24, 3.77)	0.007	-	-
Housing condition				
Unstable	Ref			
Relatively stable	3.60 (1.03, 12.61)	0.046	-	-
HIV risk behaviors in the last 3 months				
≥ 2 sex partners with anal sex	1.57 (1.07, 2.31)	0.021	1.54 (1.04, 2.29)	0.031
CRAI with male sex partners				
No	Ref		-	-
Yes	1.07 (0.71, 1.60)	0.749	-	-
HIV positive male partners				
No	Ref		Ref	
Yes	2.54 (1.04, 6.17)	0.040	2.16 (0.88, 5.32)	0.095
Not sure	1.90 (1.23, 2.92)	0.004	2.04 (1.31, 3.18)	0.002
Recreational drug use				
No	Ref		Ref	
Yes	1.62 (1.09, 2.423)	0.018	1.58 (1.05, 2.40)	0.030
History of STIs				
Syphilis Positive	1.80 (0.72, 1.93)	0.514	-	-
HSV-2 Positive	2.11 (1.29, 3.44)	0.003	2.15 (1.30, 3.57)	0.003
CROPrEP history				
Oral PrEP regimen				
D-PrEP	Ref		Ref	
ED-PrEP	0.66 (0.45, 0.97)	0.033	0.66 (0.45, 0.98)	0.037
Switch PrEP regime				
No	Ref		-	-
Yes	0.90 (0.60,1.36)	0.620	-	-
Suboptimal oral PrEP adherence				
No	Ref		Ref	
Yes	0.50 (0.31, 0.78)	0.003	0.58 (0.36, 0.94)	0.026

*Note*: *CI* confidence interval, *CNY* Chinese Yuan, *USD* United States dollar, *HIV* human immunodeficiency virus, *CRAI* condomless receptive anal intercourse, *STI* sexual transmitted infection, *HSV-2* syphilis and herpes simplex virus 2, *CROPrEP* the China Real-world Study of Oral PrEP, *PrEP* pre-exposure prophylaxis, *LAI-PrEP* long-acting injectable PrEP. -: socio-demographic characteristics were controlled for. NA: correlates that did not reach a *p*-value of > 0.2 in the univariate analysis had not been included in the multivariate analysis

### LAI-PrEP willingness among FCPs considering the oral PrEP regimen and adherence

**Fig. 2:**
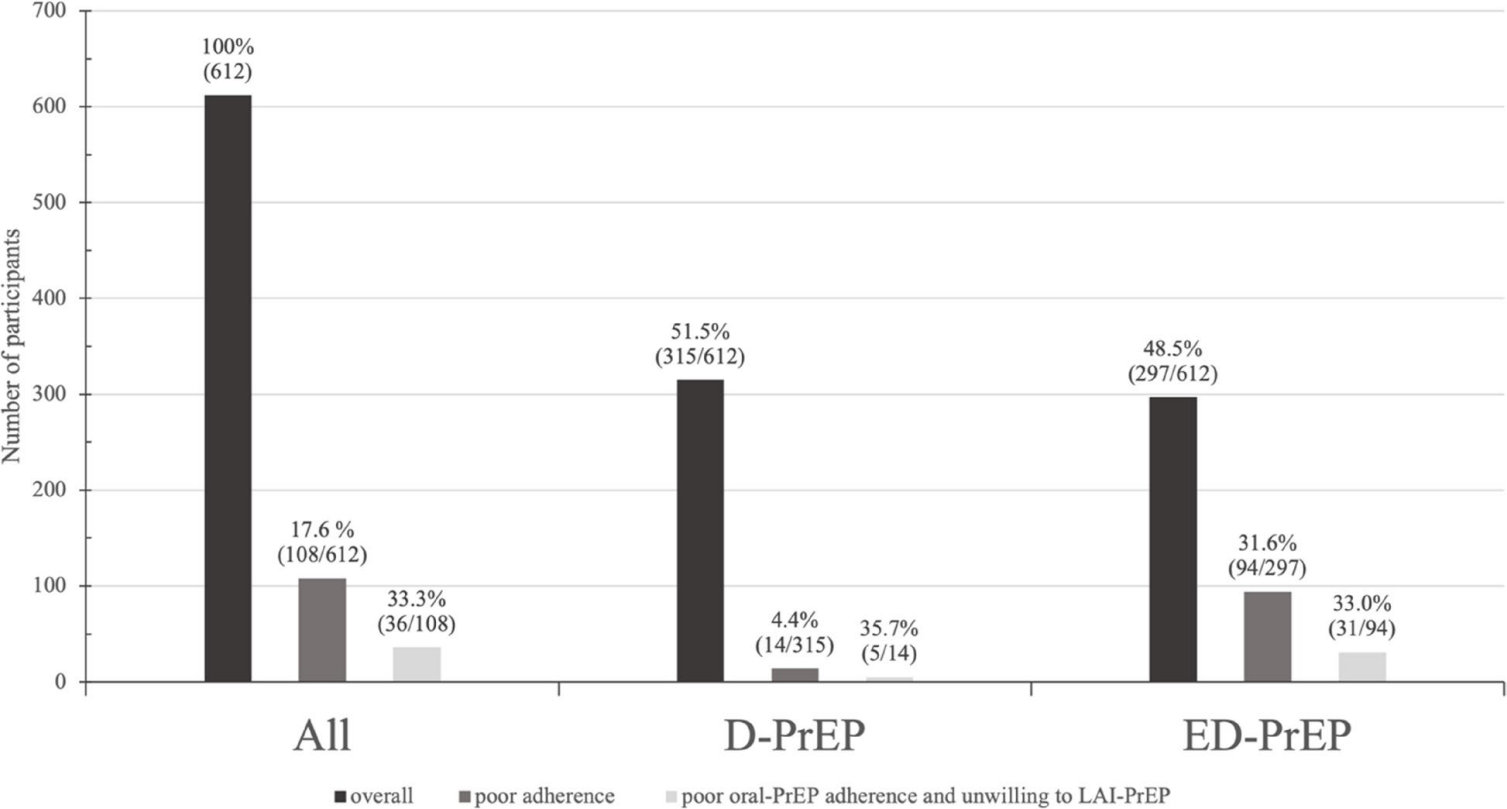
Willingness to use long-acting injectable PrEP (LAI-PrEP) among former CROPrEP participants with suboptimal daily oral-PrEP (D-PrEP) and event-driven PrEP (ED-PrEP) adherence. The three groups represent all participants, D-PrEP users, and ED-PrEP users, respectively. Each group consists of three differently colored bars. The darkest colored bars (named *overall*) represent all individuals within each group. In this study, FCPs were included including D-PrEP users and ED-PrEP users. The middle shade (named *poor adherence*) indicates those within the group with suboptimal oral PrEP adherence. And the lightest colored bars (named *poor oral-PrEP adherence and unwilling to LAI-PrPE*) represent individuals within the group with both suboptimal oral PrEP adherence and a lack of willingness to use LAI-PrEP. FCPs, former CROPrEP participants; D-PrEP, daily oral PrEP; ED-PrEP, event-driven PrEP; LAI-PrEP, long-acting injectable PrEP

During the CROPrEP project period, 108 (108/612, 17.6%) FCPs had suboptimal oral-PrEP adherence, including 14 (14/315, 4.4%) D-PrEP users and 94 (94/297, 31.6%) ED-PrEP users. Among FCPs who had suboptimal adherence, nearly one-third of former D-PrEP users (5/14, 35.7%) and ED-PrEP users (31/94, 33.0%) were unwilling to take LAI-PrEP (Fig. 2). FCPs who had suboptimal oral PrEP adherence and were unwilling to take LAI-PrEP (vs. others) were older (*P* = 0.009) and had lower education levels (*P* < 0.001), lower monthly income (*P* = 0.009), less relatively stable house conditions (*P* = 0.017), and more former ED-PrEP regimens (*P* < 0.001) than the other FCPs (Table S4 in Supplement 4).

## Discussion

This study’s findings on the willingness of former PrEP users to take LAI-PrEP considering adherence provide a valuable reference for future policy-making to promote LAI-PrEP implementation and further curb the HIV epidemic. In this study, only one-third of FCPs had heard about LAI-PrEP. After a brief introduction to LAI-PrEP, more than three-quarters of the participants were willing to take LAI-PrEP, especially former D-PrEP users, and nearly a third of the participants chose LAI-PrEP as their preferred formulation over oral PrEP. This study also showed that FCPs who had no less than two male partners, male partners with unknown HIV statuses, recreational drug use, HSV-2-positive results, former D-PrEP regimens, and good oral PrEP adherence were more likely to take LAI-PrEP. It is hoped that the new LAI-PrEP modality will break barriers to oral PrEP access, retention, and adherence. The best scenario is that LAI-PrEP can cover MSM who have suboptimal adherence to oral PrEP [14, 15].

In this study, we observed a significant lack of awareness regarding LAI-PrEP among MSM. This finding underscores a critical gap in knowledge about this emerging HIV prevention modality [27, 28], especially among MSM who have prior experience with oral PrEP. While the advantages of LAI-PrEP, such as the reduced dosing frequency and potentially enhanced adherence, hold promise for improving HIV prevention efforts, our results suggest that these benefits may not be fully realized due to low awareness. This highlights the urgent need for comprehensive education and awareness campaigns targeting both health care providers and potential PrEP users. Addressing this awareness gap is essential for optimizing the impact of LAI-PrEP and expanding its uptake among populations at risk of HIV infection.

However, this study showed that although most FCPs were willing to take LAI-PrEP and the majority had good oral PrEP adherence, nearly one-third of former PrEP users with poor adherence were unwilling to take LAI-PrEP. This study’s findings may address a gap in the research. The results indicate that LAI-PrEP, a constantly updating and evolving toolkit to offer novel strategies to increase PrEP use, may not cover the possible targeted population [29]. This study may help public health policy-makers identify the novel tool’s target population, thereby providing the opportunity to implement corresponding measures to allow LAI-PrEP to play an increasingly important role in ever-expanding PrEP use.

This study’s findings on the willingness to take LAI-PrEP and the preference for LAI-PrEP among MSM with oral PrEP experience indicate that LAI-PrEP, as an updated tool, has good prospects for HIV prevention. In this study, more than three-quarters (77.7%) of FCPs were willing to take LAI-PrEP. According to previous studies, the proportion of MSM who are willing to take LAI-PrEP ranges from 25 to 88% among different groups of MSM, and the majority of these studies show that over two-thirds of MSM are willing to take LAI-PrEP [19–21, 30–36]. Moreover, prior studies have also revealed that the willingness of MSM with oral PrEP experience to take LAI-PrEP is high [22, 23]. These results suggest that LAI-PrEP, as a potential administrative method, will be popular. For preferences for the PrEP modalities, in this study, nearly one-third (33.1%) of FCPs selected LAI-PrPE as their first choice rather than oral PrEP. This result is inconsistent with some studies reporting that LAI-PrEP is a less preferred modality than other mediations [37–39]. There are also some studies reporting that LAI-PrEP is a less preferred choice [19, 20, 22, 27]. In addition, a previous study revealed that the availability of LAI-PrEP would increase PrEP interest by 24.5% over oral PrEP alone. Taken together, LAI-PrEP may be suitable for popularization and application, and through the addition of a second PrEP modality, LAI-PrEP can yield increased PrEP population coverage.

The findings from our study, particularly regarding FCPs’ preferences related to payment for either oral PrEP or LAI-PrEP, shed light on the economic considerations that individuals may weigh when choosing a prevention strategy. The cost associated with HIV prevention methods is undeniably a critical factor in determining their accessibility and acceptability. It is crucial to recognize that cost implications can significantly influence these decisions in real-world settings. In resource-constrained environments, the financial burden of accessing PrEP, whether through oral regimens or LAI formulations, can present substantial barriers. Therefore, as we move forward with the implementation of LAI-PrEP and efforts to expand PrEP coverage, it becomes imperative to comprehensively address the affordability aspect.

This study also identified significant associations between the number of male sex partners, the HIV serological status of male sex partners, recreational drug use, HSV-2 infection status, and the willingness to take LAI-PrEP among MSM with oral PrEP experience. Previous studies reported on factors associated with MSM’ willingness to take LAI-PrEP or the preference for LAI-PrEP, including demographic (e.g., age [22, 31, 35], ethnicity [23, 33, 35, 39], educational level [21, 27, 34], income [22], and insurance [39]), behavioural (e.g., sex roles [20, 31], number of male sexual partners [34, 35], HIV serological status of male sex partners [21], condom use [19], and HIV testing [20]), HIV risk perception-related [21, 27], and PrEP-related factors (e.g., PrEP use experience [22, 27, 33, 35]). The factors, such as demographic characteristics and HIV-related behaviours, in this study were similar to those in previous studies. In this study, we found that FCPs with positive HSV-2 test results were more likely to take LAI-PrEP. HSV-2 infection has been associated with a higher risk of HIV infection in previous research [40, 41]. Individuals with HSV-2 may perceive themselves as being at a higher risk of HIV infection and, therefore, may be more motivated to seek out LAI-PrEP as a potentially more effective HIV prevention method. However, it is important to note that this study did not directly assess this vulnerability perception. Further research is needed to fully understand the motivations behind this association and the potential role of HSV-2 infection in shaping preferences for LAI-PrEP. Despite this, a prior study indicated that having previously been diagnosed with an STI showed borderline significance [34]. The result suggests that care providers in the future need to discuss sexual health when helping in decision-making regarding PrEP initiation.

To our knowledge, this study represents a novel investigation into the associations between former oral PrEP experience (including oral PrEP regimen and adherence) and the willingness to take LAI-PrEP. Daily oral PrEP provides an entirely new biological control for HIV prevention [25]. Event-driven PrEP, as an alternative method for MSM with infrequent HIV-related events, offers potential advantages such as a lower pill burden, lower financial cost, and fewer adverse effects than D-PrEP to expand PrEP coverage [6, 42]. However, conferring the protective effect of oral PrEP is a continuous process, including awareness, willingness, initiation, persistence, and adherence, which are all important limiting factors of the efficacy of oral PrEP. LAI-PrEP holds promise in addressing these barriers and has become an important tool in an ever-expanding PrEP toolkit (Supplementary 2 eFigure 1) [14, 15]. Previous studies have revealed that MSM with previous or current oral PrEP use are willing to use or switch to LAI-PrEP [22, 27, 33, 35]. This study has further shown that compared with ED-PrEP users, D-PrEP users are more willing to use LAI-PrEP. The results suggest a potential maintenance burden of daily oral PrEP and the need for LAI-PrEP as an alternative. Furthermore, this study sheds light on the potential of LAI-PrEP to improve adherence for MSM with prior oral PrEP experience, contributing novel insights to the field. In this study, compared to other studies, there were low rates of ED-PrEP adherence, which might be attributed to regional differences, health care infrastructure, access to care, and participant demographics [43]. Perhaps the most striking result is that FCPs with suboptimal adherence to oral PrEP were less willing to take LAI-PrEP, which is inconsistent with the prediction. Thus, to minimize the barriers to adherence to oral PrEP and increase PrEP uptake through LAI-PrEP, further programs should pay more attention to improving the willingness of former oral PrEP users with poor adherence to take LAI-PrEP.

This study has some strengths. This study contributes to the emerging body of research exploring LAI-PrEP as an alternative tool to address adherence challenges and expand PrEP coverage among former users. It examined the willingness of former PrEP users to take LAI-PrEP and explored associated factors, offering valuable insights into this evolving field. This study provides valuable evidence to guide future LAI-PrEP programming efforts. In addition, this study was conducted in multiple cities in China and included a diverse urban sample with more generalizable results than those provided by a single-site study.

This study also has some limitations. First, the results may have been impacted by social desirability and recall bias as participants self-reported their behaviours through the online survey. Second, there is a risk of participant selection bias because nearly a quarter of FCPs were not included in the analysis. Third, there is a lack of a gold standard for measuring adherence. Numerous methods are commonly used to evaluate oral PrEP adherence, and each possesses unique advantages and disadvantages. Fourth, the associated testing costs for LAI-PrEP may indeed serve as a barrier for individuals with low income or those who are homeless to access and use LAI-PrEP. This study specifically focused on investigating FCPs' willingness to use LAI-PrEP and did not assess the impact of testing expenses on their willingness. This is a crucial point to consider, and future research should delve into the explicit effects of covering testing costs on the promotion and adoption of LAI-PrEP. Finally, the study was conducted among MSM only in four large Chinese cities. Caution should be applied when comparing the findings to other groups, such as MSM in rural areas or those in high-risk groups, such as people who use injection drugs.

## Conclusion

Former oral PrEP users, especially daily oral PrEP users, had a high willingness to take LAI-PrEP, indicating that LAI-PrEP has good prospects for expanding PrEP coverage. However, MSM with suboptimal adherence were less likely to take LAI-PrEP. Therefore, further intervention and implementation efforts are continuously needed to improve LAI-PrEP willingness, especially for former oral-PrEP users with suboptimal adherence. STIs are also associated with the willingness to take LAI-PrEP, and sexual health should be discussed when making the decision for PrEP initiation in the future.

### Supplementary Information

**Additional file 1: Supplement 1.** Informed consent.

**Additional file 2: Supplement 2.** Online survey.

**Additional file 3: S1.** Table STROBE Statement—Checklist of items that should be included in reports of *cross-sectional studies.* **S2**. Table Checklist for Reporting Results of Internet E-Surveys (CHERRIES)^a^.

**Additional file 4: S3.** Table Characteristics of participants not included compared to those participants included. **S4.** Table Comparison between those who had suboptimal oral PrEP adherence and unwillingness to take LAI-PrEP and others. **eFigure 1.** schematic of PrEP cascade during different PrEP formulation period. PrEP eligibility refers to individuals who could have initiated PrEP. Daily oral PrEP period refers to stage after daily PrEP was firstly approved in 2012. Daily plus event-driven PrEP period refers to stage after Event-PrEP was firstly approved in 2015. Oral and LAI PrEP period refers to stage afera LAI-PrEP was firstly approved in 2020.

## Data Availability

The datasets generated and/or analyzed during the current study are not publicly available due to confidentiality policies but are available from the corresponding author on reasonable request.

## Abbreviations

CAB-LA: Long-acting injectable cabotegravir
CBO: Community-based organization
CHERRIES: The Checklist for Reporting Results of Internet E-Surveys
CI: Confidence intervals
CRAI: Condomless receptive anal intercourse
CROPrEP: The China Real-world Study of Oral PrEP
D-PrEP: Daily oral PrEP
ED-PrEP: Event-driven oral PrEP
FCPs: Former CROPrEP participants
FDA: The United State Food and Drug Administration
HIV: Human immunodeficiency virus
HSV-2: Syphilis and herpes simplex virus 2
LAI-PrEP: Long-acting injectable PrEP
MSM: Men who have sex with men
STI: Sexual transmitted infection
OR: Odds ratios
PrEP: Pre-exposure prophylaxis
