# Supplementary material for: Willingness to take long-acting injectable pre-exposure prophylaxis among men who have sex with men who participated in the CROPrEP study: a cross-sectional online study
Source: BMC Public Health. 2023 Dec 13;23:2494. doi: 10.1186/s12889-023-17325-9 (PMC10717110; doi:10.1186/s12889-023-17325-9)
Supplement: Supplementary file 1 — Additional file 1: Supplement 1. Informed consent. [file 12889_2023_17325_MOESM1_ESM.docx]

**Supplement 1** **Informed consent**

**Informed** **consent –****a** **follow-up survey study on pre-exposure prophylaxis (PrEP) and HIV self-testing to participants in CROPrEP demonstration**

Hello, welcome to participate in the project " a follow-up survey study on pre-exposure prophylaxis (PrEP) and HIV self-testing to participants in CROPrEP demonstration". It was approved by the Institutional Review Boards at the Institutional Review Board at The First Affiliated Hospital of China Medical University. This project will help determine the willingness and use conditions of PrEP as well as HIV self-testing among MSM after PrEP demonstration study. It could provide a reference for valuable advice on future intervention programming. Please read it carefully before you make the decision to join or not. The detailed description of the study purpose, procedure, benefits, risks, discomfort, and other issues were listed below. Definitely, you could withdraw from this study at any time. If you have any questions about this informed consent, please feel free to raise it to the doctor who is responsible for this study. The signature of informed consent is required if you decide to participate in this study.

**Background**

Globally, to promote PrEP-based comprehensive HIV prevention and control, 129 countries and regions worldwide have adopted World Health Organization’s (WHO) recommendation on PrEP, and 41 of them have developed and launched their national PrEP guidelines. ^18^ Moreover, 169 PrEP ongoing programs aimed to obtain first-hand evidence that would be used for the development of PrEP prevention and control measures in these countries. There is no evidence of PrEP or HIV self-testing use conditions and its correlates among MSM after PrEP demonstration study.

**Objective**

The purpose of this longitudinal study is to clarify the PrEP and HIV self-testing use conditions and its correlates among MSM after completion of PrEP demonstration. It will provide evidence for the future PrEP intervention programming, as well as policy references for relevant departments.

**Obligations**

To participate in this project, you should know and complete the following steps:

1. Provide accurate basic information and the willingness and use condition of PrEP and HIV self-testing after completion the CROPrEP demonstration.

2. This is an anonymous study, and the real name is not needed. Accurate contact information is required to ensure the continued study follow-up.

3. Voluntary to participate.

**Risks and Benefits**

**Risks**: This study does not collect any biological samples such as your blood; only questionnaire information is collected.

**Benefits**: Your participation is crucial to the contribution of promoting PrEP uptake and HIV self-testing use in China. 20 RMB ($4.2) stipends were provided after you complete the questionnaire. (Online survey to collect information, and the research staff will review the questionnaire and distribute the stipends in the form of online red envelopes. If the completion time of the questionnaire is less than 3 minutes, or there is an obvious logic error, it is considered that the questionnaire is unqualified, and the stipends will be not distributed.)

**Participation and Withdrawal**

Participation in this study is voluntary. You can refuse or withdraw from the study at any time. You will not be discriminated against, unfairly treated, or retaliated. Your medical treatment and rights will not be affected.

**Confidentiality**

It’s an anonymous survey. The privacy of personal information and participation in the study is promised and will be strictly confidential.

**Contact**

If you have questions or need help before registration, please contact:

The First Affiliated Hospital of China Medical University 86+17602406065

**Statement**

I have read this informed consent and have obtained relevant information about this study.

● I understand that I can voluntarily participate in the study without any interest deficiency or adverse consequences.

● I am willing to cooperate with the researchers to complete the study.

● I know that my personal identity and privacy will be strictly confidential.

This informed consent is made in duplicate, one of which is signed and kept by the respondents, and the other is kept confidential. (clicking the "agree" button will be regarded as your agreement to sign the " Informed consent - longitudinal study on pre-exposure prophylaxis (PrEP) use conditions to participants in CROPrEP demonstration”).

According to the research content introduced by the researcher, I have decided to participate in:

Participant signature: ____________ Date: ______________

Tel: ______________

Researcher 's statement on the implementation of informed consent

I have fully explained the purpose, process, possible risks, potential benefits of the participation in this study and answered all relevant questions of the participant with satisfaction.

Researcher signature: ____________ Date: ______________

Tel: ______________
