# Supplementary material for: Willingness to take long-acting injectable pre-exposure prophylaxis among men who have sex with men who participated in the CROPrEP study: a cross-sectional online study
Source: BMC Public Health. 2023 Dec 13;23:2494. doi: 10.1186/s12889-023-17325-9 (PMC10717110; doi:10.1186/s12889-023-17325-9)
Supplement: Supplementary file 2 — Additional file 2: Supplement 2. Online survey. [file 12889_2023_17325_MOESM2_ESM.docx]

**Supplement 2 Online survey**

**An online survey on** **pre-exposure prophylaxis (PrEP) and HIV self-testing to participants after CROPrEP demonstration (LAI-PrEP parts)**

It’s the document that contains the complete text of the English language version of the questionnaire, including the introductory text, closing remark, and subheadings. Routing instructions and notes within < > in this document did not appear in the electronic survey questionnaire. Notes within [ ] indicate the type of the questions or options. ***** means the compulsory questions. All questions have appeared in the numbered sequential order in the questionnaire.

**Standard Response Sets**

5-point agree-disagree response set

1=Strongly disagree

2=Disagree

3= Neutral

4=Agree

5=Strongly agree

**[INRODUTORY TEXT]**

Welcome to this survey on pre-exposure prophylaxis (PrEP) and HIV self-testing to participants after CROPrEP demonstration. In order to timely understand the willingness and use conditions of PrEP and HIV self-testing among MSM after PrEP demonstration study, we sincerely invite you to participate in this questionnaire survey. This investigation is anonymous and does not involve personal privacy. Your answer will provide valuable advice on future intervention programming during the PrEP demonstration stage.

Please complete this survey once only from December 2020 to January 2021. After completing the survey, we will provide a subsidy of about $3.20 per person. Thanks for your support!

You can call our staff to get more information about the survey.

Tick both of the following boxes to confirm that you have read and understood what the questionnaire is about and that you wish to take part in. [multiple choice] *****

□I have understood the above information

□I would like to take part in this survey [go to informed consent ]

**Part A. Demographic Characteristics**

**A1. Your age is…?** [Fill in the blank] *****

**A2. Highest education attained:** [single choice] *

High or below

College and greater

**A3. What is your current marital status?** [single choice] *

Currently single

Married or cohabitated with female

Cohabitated with male

**A4. What is your current average monthly income?** [single choice] *

No fixed income

Below 4,000 RMB (<$619)

At least 4,000 (≥$619)

A5. **How is your housing condition?** [single choice] *

Unstable (homeless)

Relatively stable

**Part B PrEP use condition**

**B1.** **If there are free PrEP medications, but you should pay for PrEP related routine laboratory tests, would you like to take free PrEP medications to prevent HIV infection?** [single choice] *

Yes

No

I’m not sure

**B2. If the CROPrEP group provides free counseling service, would you like to pay for oral PrEP medications and related routine laboratory to prevent HIV infection?** [single choice] *

Yes

No

I’m not sure

**B3.** <if B2= “Yes”>**How much of the highest cost are you willing to pay for PrEP medications monthly?** [Fill in the blank] *****

**Note**: Reference prices of oral PrEP medications market price that we obtained online：Truvada^®^-1980 RMB a bottle, Glyke^®^ (Chinese genic medications)-1180 RMB a bottle, Indian genic medications-200 to 500 RMB a bottle.30 capsules each bottle.

**B4. Do you pay for oral PrEP medications after completion of the CROPrEP study?** [single choice] *

Yes

No

**B4. Have you heard about long-acting injectable PrEP?** [single choice] *

Yes

No

I’m not sure

**Note:< if B4= “No” or “Not sure”>** Long-acting injectable (LAI) PrEP is a new alternative to oral PrEP. . A previous study revealed that intramuscularly long-acting injectable cabotegravir (CAB-LA) with every 8 weeks was superior to daily oral TDF-FTC in preventing HIV infection among MSM. And in 2021 December the United State Food and Drug Administration (FDA) approved LAI-PrEP for HIV prevention.

**B5. If free LAI-PrEP was available, but you had to pay for PrEP-related routine laboratory tests, would you take LAI-PrEP to prevent HIV infection?** [single choice] *

Yes

No

I’m not sure

**B6.**  **If the CROPrEP group provided free counselling services, would you pay for LAI-PrEP medications and related routine laboratory tests to prevent HIV infection?** [single choice] *

Yes

No

I’m not sure

**B7 What is your preference for the two different PrEP modalities?** [single choice] *

LAI-PrPE

Oral-PrEP

The more effective way

Either

None

I’m not sure

**Quality control question1:** You should only choose the option of “Completely agree” [single choice] *

Completely agree

Basically agree

Disagree

**Part C. HIV-related risk behaviors**

**C1. How many anal sexual partners have you had in the past three months?** [Fill in the blank] *****

**C2. Have you ever had condomless receptive anal intercourse with male partners in the past three months?** [single choice] *<filling in a number≥0.>

Yes

No

**C3. Have you had HIV-positive male partners in the past three months?** [single choice] *

Yes

No

I’m not sure

**C4. In the past three months, have you used the following** **chemsex-related drugs?** [single choice] *

**Note**: chemsex-related drugs such as Inhalants (RUSH, popper, nitrogen, glue, olfactory oil, etc.)**,** Amphetamine-type stimulants (Dalibor, methamphetamine, etc.), Ketamine, Cannabis (cannabis, cannabis buds, hemp grass, cannabis resin, etc.), Tramadol/ Dextromethorphan Hydrobromide Tablets**,** Codeine phosphate**,** Hallucinogens (Ecstasy, Zero capsule, psychedelic mushrooms, trips, K powder, etc.)**,** 5-MeO-Dip**,** Nitrous oxide and so on.

Yes, I have had some of above

No, I have had none of above

**Quality control question2: What's the weather today?** [single choice] *

Sunny

Cloudy

Raining

Snowing

Sunday

Thanks for your participation. Now please press 'submit'. Once you submitted the questionnaire, you no longer has repeat access to the questionnaire.
