# Supplementary material for: Willingness to take long-acting injectable pre-exposure prophylaxis among men who have sex with men who participated in the CROPrEP study: a cross-sectional online study
Source: BMC Public Health. 2023 Dec 13;23:2494. doi: 10.1186/s12889-023-17325-9 (PMC10717110; doi:10.1186/s12889-023-17325-9)
Supplement: Supplementary file 4 — Additional file 4: S3. Table Characteristics of participants not included compared to those participants included. S4. Table Comparison between those who had suboptimal oral PrEP adherence and unwillingness to take LAI-PrEP and others. eFigure 1. schematic of PrEP cascade during different PrEP formulation period. PrEP eligibility refers to individuals who could have initiated PrEP. Daily oral PrEP period refers to stage after daily PrEP was firstly approved in 2012. Daily plus event-driven PrEP period refers to stage after Event-PrEP was firstly approved in 2015. Oral and LAI PrEP period refers to stage afera LAI-PrEP was firstly approved in 2020. [file 12889_2023_17325_MOESM4_ESM.docx]

Supplement 4

S3 Table Characteristics of participants not included compared to those participants included.

| Characteristics | Participants  N=612 | Non-Participants  N=292 | P |
| --- | --- | --- | --- |
| socio-demographic characteristics |  |  |  |
| Age, mean (SD), y | 34.54 (8.75) | 34.39 (8.39) | 0.166 |
| Education level |  |  | 0.294 |
| High school or less | 108 (17.6) | 60 (20.5) |  |
| College and greater | 504 (82.4) | 232 (79.5) |  |
| Monthly personal income, CNY (USD) |  |  |  |
| No fixed income | 69 (11.3) | 19 (6.5) | <0.001 |
| Below 4,000 (619) | 159 (26.0) | 49 (16.8) |  |
| At least 4,000 (619) | 384 (62.7) | 224 (76.7) |  |
| Housing condition |  |  | 0.392 |
| Unstable | 10 (1.6) | 2 (0.7) |  |
| relatively stable | 602 (98.4) | 290 (99.3) |  |
| History of STIs |  |  |  |
| Syphilis Positive | 120 (19.6) | 51 (17.5) | 0.442 |
| HSV Positive | 166 (27.1) | 69 (23.6) | 0.263 |
| CROPrEP history |  |  |  |
| Oral PrEP regimen |  |  | 0.040 |
| Daily | 315 (51.5) | 129 (44.2) |  |
| Event-driven | 297 (48.5) | 163 (55.8) |  |
| Switched oral PrEP regimen |  |  | 0.005 |
| No | 429 (70.1) | 177 (60.6) |  |
| Yes | 183 (29.9) | 115 (39.4) |  |
| Suboptimal oral PrEP adherence |  |  | 0.846 |
| No | 504 (82.4) | 242 (82.9) |  |
| Yes | 108 (17.6) | 50 (17.1) |  |

S4 Table Comparison between those who had suboptimal oral PrEP adherence and unwillingness to take LAI-PrEP and others

|  | Overall  N=612 | Oral PrEP poor adherene and LAI-PrEP unwillingness  N=36 (%) | Others  N=576 (%) | P values |
| --- | --- | --- | --- | --- |
| socio-demographic characteristics |  |  |  |  |
| Age groups (years) |  |  |  | 0.009 |
| 18-29 | 243 (39.7) | 14 (38.9) | 229 (39.8) |  |
| 30-49 | 326 (53.3) | 15 (41.7) | 311 (54.0) |  |
| 50 or above | 43 (7.0) | 7 (19.4) | 36 (6.3) |  |
| Education level |  |  |  | <0.001 |
| High school or less | 108 (17.6) | 15 (41.7) | 93 (16.1) |  |
| College and greater | 504 (82.4) | 21 (58.3) | 483 (83.9) |  |
| Monthly personal income, CNY (USD) |  |  |  |  |
| No fixed income | 69 (11.3) | 7 (19.4) | 62 (10.8) | 0.009 |
| Below 4,000 (619) | 159 (26) | 15 (41.7) | 144 (25/0) |  |
| At least 4,000 (619) | 384 (62.7) | 14 (38.9) | 370 (64.2) |  |
| Housing condition |  |  |  |  |
| Unstable | 10 (1.6) | 3 (8.3) | 7 (1.2) | 0.017 |
| relatively stable | 602 (98.4) | 33 (91.7) | 569 (98.8) |  |
| HIV-related behaviors in the past 3 months |  |  |  |  |
| ≥2 sex partners with anal sex | 359 (58.7) | 16 (44.4) | 343 (59.5) | 0.074 |
| CRAI with male sex partners | 205 (33.5) | 11 (30.6) | 194 (33.7) | 0.700 |
| HIV positive male sexual partners |  |  |  | 0.127 |
| No | 351 (57.4) | 25 (69.4) | 326 (56.6) |  |
| Yes | 47 (7.7) | 0 | 47 (8.2%) |  |
| Not sure | 214 (35.0) | 11 (30.6) | 203 (35.2) |  |
| Recreational drug use | 257 (42.0) | 10 (27.8) | 247 (42.9) | 0.075 |
| History of STIs |  |  |  |  |
| Syphilis Positive | 120 (19.6) | 3 (8.3) | 117 (20.3) | 0.079 |
| HSV Positive | 166 (27.1) | 5 (13.9) | 161 (28.0) | 0.066 |
| CROPrEP history |  |  |  |  |
| Oral PrEP regimen |  |  |  | <0.001 |
| Daily | 315 (51.5) | 5 (13.9) | 310 (53.8) |  |
| On-demand | 297 (48.5) | 31 (86.1) | 266 (46.2) |  |
| Switched oral PrEP regimen | 183 (29.9) | 5 (13.9) | 178 (30.9) | 0.031 |


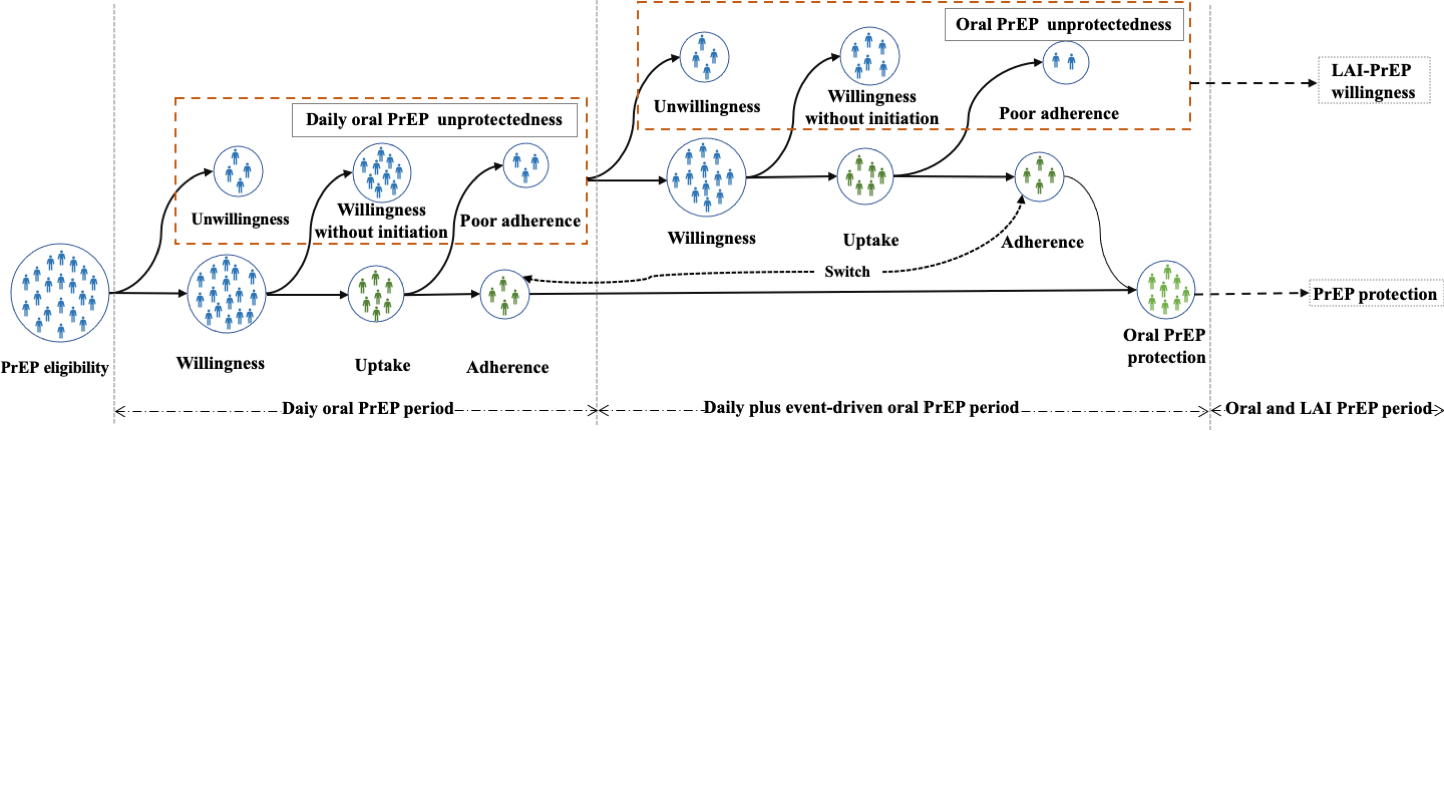


eFigure 1 schematic of PrEP cascade during different PrEP formulation period. PrEP eligibility refers to individuals who could have initiated PrEP. Daily oral PrEP period refers to stage after daily PrEP was firstly approved in 2012. Daily plus event-driven PrEP period refers to stage after Event-PrEP was firstly approved in 2015. Oral and LAI PrEP period refers to stage afera LAI-PrEP was firstly approved in 2020.
